# Inflammatory Pseudotumour of the Lung: A Case Report and Literature Review

**DOI:** 10.1155/2012/214528

**Published:** 2012-12-05

**Authors:** R. Morar, A. Bhayat, G. Hammond, H. Bruinette, C. Feldman

**Affiliations:** ^1^Division of Pulmonology, Department of Medicine, University of the Witwatersrand, Johannesburg, South Africa; ^2^Thoracic Surgery Department, University of the Witwatersrand, Johannesburg, South Africa; ^3^Ampath Laboratories, Johannesburg, South Africa

## Abstract

We describe a patient with inflammatory pseudotumour of the lung. He was a young man who presented with haemotysis and the chest X-ray and computerized tomography were indicative of a nonbenign lesion in the right upper lobe. Excision biopsy confirmed the diagnosis of inflammatory myofibroblastic pseudotumour of the lung. This is a rare inflammatory nonneoplastic condition commonly affecting children and young adults.

## 1. Introduction

Inflammatory pseudotumour of the lung is a rare condition. It is an inflammatory, reactive, and nonneoplastic process characterized by unregulated growth of inflammatory cells. It occurs most commonly in children and young adults and is usually found incidentally. Inflammatory pseudotumour may mimic lung cancer and thereby pose a diagnostic dilemma for the clinician. We recently managed a case that we describe, as well as review the literature.

## 2. Case Presentation

**Figure 1:**
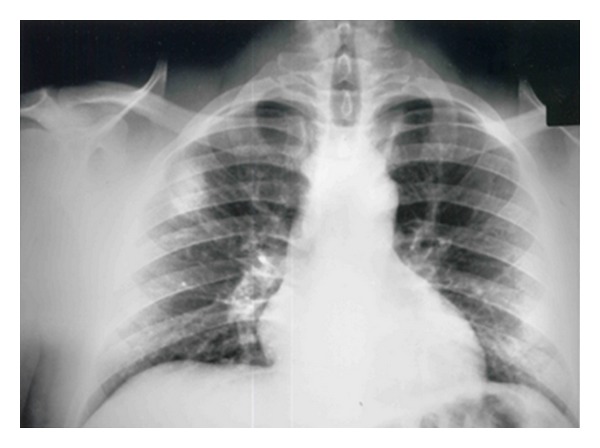
Chest X-ray revealing a rounded opacity in the right upper lobe.

A 28-year-old nonsmoking male was referred for diagnostic evaluation of recurrent mild haemoptysis associated with a right upper lobe solitary pulmonary nodule of 8 weeks duration. Chest radiograph revealed a 1.5 × 2 cm coin lesion in the posterior segment of the right upper lobe of the lung (Figure 1).

**Figure 2:**
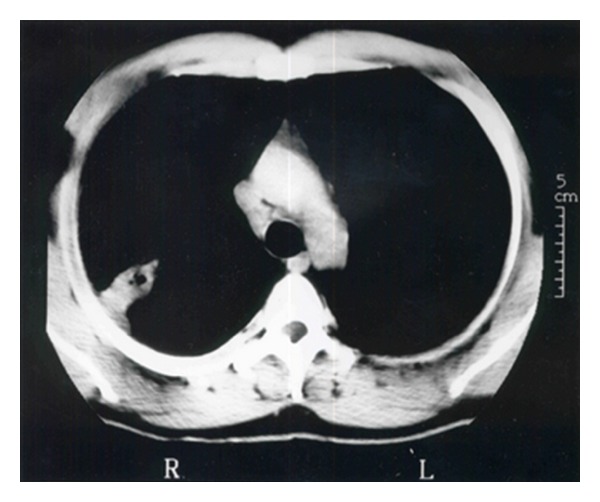
CT scan of the chest demonstrating the lesion in the posterior segment of the right upper lobe with central lucency.

The medical history was noncontributory. A computed tomographic (CT) scan of the chest confirmed the chest radiograph findings; a solid mass was noted in the posterior segment of the right upper lobe of the lung with an area of central lucency (Figure 2).

There was no hilar lymphadenopathy. Sputum microscopy, culture, and cytological examination were noncontributory. The ESR was 14, the haemoglobin 14.6 g/dL, and the leukocyte count of 8.8 × 10^9^/L. The other serum haematological and biochemical results were normal.

In view of the patient's ongoing haemoptysis and lack of response to antibiotics he was subjected to an exploratory thoracotomy and wedge resection of the lesion in the right upper lobe. A solid mass in the posterior segment of the right upper lobe was noted and it was excised. No associated lymphadenopathy was noted. Frozen section of the mass revealed no evidence of carcinoma or tuberculosis. The patient developed a right upper lobe lung abscess postoperatively, and underwent a complete right upper lobe lobectomy.

**Figure 3:**
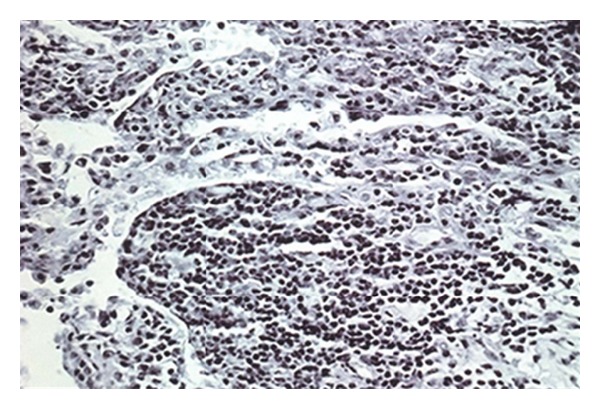
Photomicrograph demonstrating intra-alveolar myofibroblastic proliferation (organising pneumonia-like type).

Macroscopically, a well-circumscribed tan-coloured nodule measuring 35 × 15 mm was present. The cut surfaces of the tumour revealed a haemorrhagic necrotic centre. Microscopically, the mass consisted of a heavy inflammatory cell infiltrate composed predominantly of lymphocytes, with plasma cells and histiocytes. Foamy histiocytes with macrophages were also seen, as well as occasional eosinophils and neutrophils. Focal areas of micro-abscess formation with necrosis were also noted. A marked degree of fibrosis of the interstitial tissue was present with proliferating myofibroblasts. The histological characteristics were compatible with an inflammatory myofibroblastic pseudotumour (organising pneumonia-like type; Figure 3).

The postoperative course was uneventful, the patient was discharged from the hospital one week later, and three years after surgical resection the patient remains well and free of disease.

## 3. Discussion

Inflammatory pseudotumours of the lung are rare and were first described in the lung in 1939 [1]. They are not limited to the lung and can grow in any organ such as the brain, liver, spleen, lymph nodes, salivary glands, breast, soft tissues and skin [2]. Although inflammatory pseudotumours are regarded as inflammatory or reactive lesions rather than neoplasms, they may have features such as local invasion or recurrence, distant metastases, and cytogenetic clonal changes [3–6]. They have an increased predilection to develop in children [7, 8]. The aetiology and pathogenesis remain uncertain. There are several theories, most of which postulate an unchecked or exaggerated immunologic response to a viral or foreign antigen-antibody reaction [9]. Inflammatory pseudotumours typically consists of variable amounts of stromal and cellular elements, with the myofibroblast, a cell involved in tissue repair, recognized as the principal cell-type [3, 10–12]. Depending on the major histopathologic features, inflammatory pseudotumours are divided into the following types: fibrous histiocytoma, lymphoplasmacytic, and organising pneumonia. Because of their variable histology, these masses have several synonyms, including plasma cell granuloma, myofibroblastic tumour, xanthoma, xanthogranuloma, xanthomatous pseudotumour, and plasma cell histiocytoma [2, 4, 9].

Inflammatory pseudotumours are usually considered to be benign tumours, principally occurring in younger patients. They are the most common isolated primary tumour of the lung in children younger than 16 years [8]. More than half of patients are less than 40 years of age [1, 2, 6]. Such lesions account for less than 1% of all lung tumors with no gender or race preponderance [1, 3, 4]. Many patients are asymptomatic, with inflammatory pseudotumours discovered incidentally on examination of chest radiographs. Weight loss, fever, and fatigue have been also described [1]. If the patients are symptomatic, cough, fever, weight loss, fatigue, haemoptysis, dyspnoea, clubbing, chest pain, and arthralgias may be noted [1–4, 13]. No specific findings on physical or laboratory examinations exist.

Radiological examination usually demonstrates a solitary peripheral nodule or mass of 1 to 10 cm in diameter and the lesions are typically peripheral and in the lower lobes [6]. Multiple lung masses, pneumonic consolidation, atelectasis, hilar or mediastinal masses, pleural effusion, and cavitation are unusual [4–6]. Radiographic images and invasive diagnostic procedures, including bronchoscopy and percutaneous fine needle aspiration biopsy, are considered insufficient for diagnosis. Consequently, surgery is crucial for both diagnostic and therapeutic reasons and frozen section histological examination is also subject to errors [1, 3, 9, 13–15].

Macroscopically, inflammatory pseudotumours are well-circumscribed, non-encapsulated, firm, usually yellow-white masses containing variable inflammation, haemorrhage, calcification, and rarely cavitation. Most are parenchymal but some are endobronchial and may cause airway obstruction. Less than 5% invade the mediastinum and/or chest wall. Local recurrence is attributed to incomplete resection of the primary lesion. Metastasis of the tumor to mediastinum or the brain even many years after complete resection has been described. Rarely, simultaneous intra- and extrathoracic locations may occur. Association with other malignancies in sporadic cases has been reported [3, 4, 13].

Microscopically, the lesions consist of variable mixtures of fibroblasts and granulation tissue, fibrous tissue, and inflammatory cells including lymphocytes, histiocytes, giant cells, macrophages, neutrophils, eosinophils, and typically large numbers of plasma cells [1, 3, 4]. Immunohistochemistry has demonstrated the polyclonal nature of plasma cells with immunoglobulin G predominance [16]. Frozen sections performed at the time of operation are often indeterminate [1, 2, 13]. The differential diagnosis includes lymphoma, sarcoma, and fibrosis [3, 4, 12]. Usually the pathologist is able to eliminate a neoplastic process. If uncertainty remains on frozen section and if the mass can be safely removed, complete resection is recommended.

Complete surgical resection of the inflammatory pseudotumour remains the best treatment, to exclude malignancy and to achieve cure [3, 4, 9, 13–15]. Wedge resection is adequate treatment if removal is complete. Lobectomy should be performed if it is required for complete resection and if the patient's pulmonary reserve is adequate. Non-surgical treatment modalities including radiotherapy, chemotherapy, and corticosteroids may have a place in the setting of incomplete surgical resection, multifocal disease, postoperative tumour recurrence, or contra-indications to lung resection [3, 4, 13, 16–20]. Although spontaneous regression may occur, local expansion may cause significant morbidity and occasional death. The prognosis of these rare tumours is excellent after complete surgical excision. There is a low incidence of recurrence with long-term follow-up after complete removal of the mass [3, 4, 13, 17, 21]. Patients with recurrent disease should undergo re-resection [1, 4, 14, 17].

## 4. Conclusion

Although inflammatory pseudotumours of the lung are rare and the most common clinical picture is one of an asymptomatic, well-circumscribed lung mass that mimics cancer, clinicians need to bear in mind the diverse clinical presentations. Surgical excision is usually indicated to reach a firm diagnosis and cure. As preoperative investigation is not diagnostic, excision of the mass is imperative in order to exclude malignancy. Complete resection, when possible, is safe and leads to excellent survival and remains the key to prevent recurrence.
